# Body Composition Results of Caucasian Young Normal Body Mass Women in the Follicular Proliferative Phase, Measured for the Different Positions of Limbs

**DOI:** 10.3390/ijerph181910214

**Published:** 2021-09-28

**Authors:** Dominika Głąbska, Agata Wojciechowska, Karolina Cackowska, Dominika Guzek

**Affiliations:** 1 Department of Dietetics, Institute of Human Nutrition Sciences, Warsaw University of Life Sciences (SGGW-WULS), 159C Nowoursynowska Street, 02-776 Warsaw, Poland; agata_wojciechowska@sggw.edu.pl (A.W.); karolina_cackowska@sggw.edu.pl (K.C.); 2 Department of Food Market and Consumer Research, Institute of Human Nutrition Sciences, Warsaw University of Life Sciences (SGGW-WULS), 159C Nowoursynowska Street, 02-776 Warsaw, Poland; dominika_guzek@sggw.edu.pl

**Keywords:** body composition, bioelectrical impedance, validation, reproducibility, muscle mass, body cell mass, water content, extracellular water, intracellular water

## Abstract

The bioelectrical impedance analysis (BIA) became a standardized technique for assessing body composition, but many factors affect the reproducibility of measurement, including body and limbs position. In spite of the fact that it is recommended for patient to be in a supine position, with arms abducted at least 30° and legs abducted at approximately 45°, a lot of authors conduct their measurements with arms and legs of patients separated to not touch the body but not strictly following the recommendations. Taking this into account, the aim of the study was to analyze the body composition results of Caucasian young normal body mass women in the follicular proliferative phase, measured for the different positions of limbs in order to compare the results obtained in the recommended position (with arms abducted at least 30° and legs abducted at approximately 45°) and in the commonly used position (not following strictly the recommendations). The study was conducted in a homogenous group of 100 adult females under the age of 30 years using BIA 101/ASE with the Bodygram Pro software and its equations by Akern Srl, Firenze, Italy, based on the measurement recommendations. The measurements were conducted (1) in a recommended position of arms abducted at least 30° and legs abducted at approximately 45° and (2) with arms spread and legs separated to not touch the body to compare the body composition assessment (fat mass, fat-free mass, body cell mass, muscle mass, water content, extracellular water content, and intracellular water content). It was stated that the results obtained for various positions of limbs were positively correlated (*p* < 0.0001; R > 0.5). At the same time, the statistically significant differences dependent on the position were observed for the calculated results of body cell mass (lower results for the recommended position for the results observed in kg and % of body mass; *p* = 0.0165 and *p* = 0.0075, respectively) and muscle mass (lower results for the recommended position for the results observed in kg and % of body mass; *p* = 0.0025 and *p* = 0.0011, respectively), as well as extracellular and intracellular water (higher % of total body water for the extracellular water and lower for intracellular water; *p* = 0.0049 and *p* = 0.0115, respectively), resulting from the measured resistance and reactance values. For all listed comparisons of significantly differing variables, weighted κ statistics indicated moderate agreement (values of 0.41–0.60), and the Bland–Altman plot analysis indicated no agreement (Bland–Altman index of >5%). While compared with the reference values, the major differences were observed for extracellular/intracellular water content, as, while applying a method with arms and legs separated to not touch the body (not recommended position), the extracellular water content was underestimated for 31% and intracellular water content was overestimated for 28% of participants. It may be concluded that the recommended body position of arms abducted at least 30° and legs abducted at approximately 45° should be chosen to ensure the reliability of the BIA measurements, as, while the recommendations of a body position are not followed, the results obtained may be misleading and may not reflect the actual body composition.

## 1. Introduction

There are many methods of assessing the composition of the human body [1]. The most accurate methods include densitometry, computed X-ray tomography (CT), magnetic resonance imaging (MRI), and dual-energy X-ray absorptiometry (DXA) [2]. However, these methods are recommended mainly for research purposes, because the measurements are expensive and may be associated with the other disadvantages, as in case of CT which produces dose radiation exposure [3].

Taking this into account, rather indirect methods are used in a clinical practice, including anthropometry and bioelectrical impedance analysis (BIA) [4]. While anthropometry is a basic method that does not allow deepen assessment, BIA is a method that allows the prediction of body composition [5], taking into account that properties of fat-free mass are measured and body fatness is calculated using specific regression equations [6]. BIA is based on the measurement of resistance of individual tissues of the body to low-intensity electric current flowing through them [7].

BIA became a standardized technique for assessing the body composition, because it provides more accurate estimates than anthropometric methods [8], being conducted while using a relatively inexpensive, portable, and easy-to-use tools [9]. This method is currently used to assess the body composition in many diseases, including those characterized by the loss of body weight, decrease in muscle mass, and cachexia, which require constant body composition monitoring [10], as well as other diseases characterized by fluid and electrolyte imbalances, which require adjusting applied therapy to the current condition [11]. At the same time, the recent systematic review by Haverkort et al. [12] confirmed that BIA may be also used for surgical and oncological patients when the measurements are performed longitudinally and under the same standard conditions. Moreover, the systematic review by Talma et al. [13] suggested that BIA is a practical method to estimate the body fat in children and adolescents, and the systematic review by Lyons-Reid et al. [14] emphasized that this method allows to predict the body composition of infants and young children at least, as well as other body composition tools.

Many factors that affect the reproducibility of BIA results are indicated, as they should be taken into account during measurements to not interfere [15]. Kyle et al. [15] listed, among other recommendations, those that should be followed: being in a fasting state, voiding bladder, avoiding physical exercise, proper electrodes, and body and limb positions.

Regarding body and limb positions, both recommendations by Kyle et al. [15] and the practical guide by Walter-Kroker [10] define that a patient should be in a supine position, with arms abducted at least 30°, as well as legs abducted at approximately 45°. However, there is a serious problem associated with the fact that a lot of authors conduct their studies while the position of their patients is not described as the recommended, and there is a serious risk of bias associated with the improper position of limbs of participants. The position of limbs of participants in a lot of studies is described as, e.g., supine position with arms spread apart from the body and legs separated [16], legs and arms extended [17], extremities in a relaxed position not touching the body [18], or legs slightly apart, and the arms not touching the torso [19]. Such positions are not according to the previously indicated recommendations [10,15], so it may be supposed that measurements conducted in such conditions may provide biased results. Taking this into account, the aim of the study was to analyze the body composition results of Caucasian young normal body mass women in the follicular proliferative phase, measured for the different positions of limbs in order to compare the results obtained in recommended position (with arms abducted at least 30° and legs abducted at approximately 45°) and in the commonly used position with arms and legs separated to not touch the body (not following strictly the recommendations). The hypothesis of the study was that, while comparing the results obtained in the recommended position (with arms abducted at least 30° and legs abducted at approximately 45°) and in the commonly used position (with arms and legs separated to not touch the body), the statistically significant differences of the body composition results will be noted, caused by the differences of the measured resistance and reactance.

## 2. Materials and Methods

### 2.1. Ethical Statement

This study was conducted in the Dietary Outpatient Clinic of the Institute of Human Nutrition Sciences, Warsaw University of Life Sciences (WULS-SGGW). It was conducted according to the guidelines of the Declaration of Helsinki and based on the approval of the Bioethical Commission of the National Food and Nutrition Institute in Warsaw (no. 0701/2015). All participants provided their informed consent to participate in the study.

### 2.2. Studied Population

The studied cohort was recruited based on the invitation distributed via social media, while student organizations from the Faculty of Human Nutrition of the Warsaw University of Life Sciences (WULS-SGGW) participated in gathering of the participants (purposive sampling with the snowball effect).

The inclusion criteria were the following:

− Females;
− Age 18–30 years;
− Caucasian ethnicity;
− Current Body Mass Index (BMI) of 18.5–25.0 kg/m^2^;
− Stable body mass declared within the previous 6 months;
− Living in Warsaw (being able to participate in the study conducted in the Dietary Outpatient Clinic of the Institute of Human Nutrition Sciences).

The exclusion criteria were the following:

− Irregular menstrual cycle declared;
− Amenorrhea declared;
− Applied hormonal contraception declared;
− Epilepsy;
− Having pacemakers or other simulators implanted;
− Having orthopedic prosthesis or other metal implants;
− Having visually abnormal body; limb; or trunk build (after serious surgical procedures and resections, after limb amputations, with serious scoliosis, etc.);
− Practicing sports professionally;
− Being pregnant or during lactation;
− Any metabolic disorders or other chronic diseases declared;
− Current undergoing body mass reduction declared;
− Current being on any special diet declared.

The participants of the study were planned to be invited for the measurements in the follicular proliferative phase of the menstrual cycle (6th to 11th day of their menstrual cycle), so this issue was not formulated within inclusion/exclusion criteria, but the study protocol set this issue within the measurement procedure.

### 2.3. Measurements

The BMI of the participants was verified based on the measurement of the weight and height. It was conducted while using an electronic medical weighing scale and a stadiometer based on the commonly applied rules, with an accuracy of 0.5 cm and 0.1 kg. The BMI was calculated based on the commonly applied equation [20].

The body composition analysis was conducted based on the bioelectrical impedance measurement. In order to provide valid and comparable measurements, each participant had the measurements conducted in the follicular proliferative phase of the menstrual cycle, defined as 6th–11th days of their menstrual cycle, as it is commonly applied [21,22], and during the same season (autumn). At the same time, all the measurements were conducted while using the same device, by the same operator, and in identical conditions.

Each participant received the information about preparation to the study and was able to participate in the study only if she followed all the rules, in order to avoid the influence of the phase of the menstrual cycle [23] or conditions of measurements on the results obtained [15]. Participants were informed to avoid any alcoholic beverage, coffee, and other caffeine beverages; avoid any physical training; and to be fasting during the measurements (not eating and not drinking for 8 h before measurements) [24], as well as to urinate 30 min before the measurement and defecate on the day of the measurements, as within the previous study [25].

All the measurements were conducted in the morning, to allow participants to be in a fasting state. During measurements, participants were in a light underwear without any metal elements (panties and soft cup bra) after taking off their other clothes, shoes, and jewelry. The measurements were conducted in a supine recumbent position, and to provide greater accuracy, the position was kept for 5 min before the measurements, to favor the balance of body fluids [26]. During measurements, participants were lying on a polyurethane foam matte (two layers) and a fabric matte (additional layer) isolation from the floor, without any metal or conductive elements.

For each participant, the measurements were conducted twice, in various positions, while the random order of the measurements was planned. The following positions were applied:

− with arms abducted at least 30°, as well as legs abducted at approximately 45°, as commonly recommended [10,15];
− with arms and legs separated to not touch the body (but not achieving 30°/45°, which are recommended [10,15]), as it is commonly applied [16,17,18,19].

The measurements were conducted on the dorsal part of right hand and right feet on a skin without any skin lesions at the location of the electrodes. Before the measurements, skin was rubbed with medical disinfection cotton pads, and the electrodes were placed on a dried skin surface. The standard Ag-AgCl Pro-Tab, PT 2334, Bio Protech electrodes (rectangular shape; contact area higher than 4 cm^2^) were used, and a distance between electrodes of at least 5 cm was kept [27]. Each participant had the measurements conducted with a frequency of 50 kHz while using the single-frequency device BIA 101/ASE (Akern Srl, Firenze, Italy) (tetrapolar electrode configuration), and two measurements (in various positions) were conducted with 5-min intervals.

During measurements, the resistance and reactance values were recorded in the moment when they remained stable. Afterwards, Bodygram Pro software was used (Akern Srl, Firenze, Italy) with its dedicated equations of the manufacturer (Akern BodyGram Pro 3.0) specific for the device to calculate the following parameters: fat mass, fat-free mass, body cell mass, muscle mass, water content, extracellular water content, and intracellular water content. These validated equations were derived from previous research and developed to be used with the dedicated Bodygram Pro software, while the applied software and equations were developed for exclusive use with BIA 101/ASE (Akern Srl, Firenze, Italy), and no other solutions should be chosen. All the parameters were expressed as % of body mass/% of water content and as kg. In various groups of patients, the bioimpedance analysis was proven to be characterized by good or acceptable reliability, validity, and sensitivity for the assessment of fat mass [28], fat-free mass [29], body cell mass [30], muscle mass [31], and water content [32].

### 2.4. Statistical Analysis

The normality of distribution was verified while using Shapiro–Wilk test. The data were compared while using the Student’s *t*-test (for parametric distributions) and Mann–Whitney *U* test (for nonparametric distributions). The analysis of correlation was conducted while using Pearson’s correlation coefficient (for parametric distribution) and Spearman’s rank correlation coefficient (for nonparametric distribution). Additional analyses included cross-classification quartiles accompanied by weighted κ statistics with linear weighting interpreted based on a commonly applied criteria: values <0.20 were interpreted as a slight agreement, 0.21–0.40—as a fair agreement, 0.41–0.60—as a moderate agreement, 0.61–0.80—as a substantial agreement, and 0.81–1.0—as an almost perfect agreement [33]. Moreover, an analysis of the Bland–Altman plots was conducted, while a Bland–Altman index of ≤5% (attributed to ≥95% of individuals observed to be within the limits of agreement) was interpreted as an agreement between the compared measurements [34].

The statistical analysis was conducted while using Statistica 8.0 (StatSoft Inc., Tulsa, OK, USA) and Bland–Altman Statistica macro by Matt Coates 2009 (StatSoft Inc., Tulsa, OK, USA), while *p* ≤ 0.05 was interpreted as significant.

## 3. Results

Based on the described inclusion and exclusion criteria, a sample of 100 participants was recruited to participate in the study. They were characterized by ages of 18–29 years (22.70 ± 2.12 years; median of 23.0 years; nonparametric distribution), and BMI of 20.83 ± 1.71 kg/m^2^ (median of 20.54 kg/m^2^; varied from 18.53 to 24.96 kg/m^2^), as described previously [25]. The weights of the studied participants were 59.26 ± 6.86 kg, and the heights of the studied participants were 168.45 ± 6.36 cm.

The comparison of the body composition results in the studied group of Caucasian young normal body mass women measured in the follicular phase in various positions (with arms abducted at least 30° and legs abducted at approximately 45°; with arms and legs separated to not touch the body) is presented in Table 1. It was stated that the statistically significant differences dependent on the position were observed for the body cell mass results (lower results for the recommended position both for the results observed in kg and % of body mass; *p* = 0.0165 and *p* = 0.0075, respectively) and muscle mass results (lower results for the recommended position both for the results observed in kg and % of body mass; *p* = 0.0025 and *p* = 0.0011, respectively), as well as extracellular and intracellular water results (higher % of total body water for the extracellular water and lower for intracellular water; *p* = 0.0049 and *p* = 0.0115, respectively).

The analysis of the correlations of the body composition results in the studied group of Caucasian young normal body mass women measured in the follicular phase in various positions (with arms abducted at least 30° and legs abducted at approximately 45°; with arms and legs separated to not touch the body) is presented in Table 2. It was stated that the results obtained for various positions of the limbs were positively correlated (*p* < 0.0001; R > 0.5).

For the variables that were associated with statistically significant differences dependent on the position, a detailed analysis of the quartile distribution of the results was conducted.

The analysis of the quartile distribution of the body cell mass results in the studied group of Caucasian young normal body mass women measured in the follicular phase in various positions (with arms abducted at least 30° and legs abducted at approximately 45°; with arms and legs separated to not touch the body) is presented in Table 3. It was stated that the results for two measurements were compatible (participants classified into the same quartile) for 59% and 58% of the studied group for the results observed in kg and the % of body mass, respectively. It corresponded the weighted κ statistics of 0.52 and 0.536, respectively, being interpreted as a moderate agreement. At the same time, while compared with the reference values, 86% of participants were classified into the same category independently of the position (results lower/higher than the reference values), and while applying a method with arms and legs separated to not touch the body (not recommended position), 8% of participants were underestimated and 6% were overestimated.

**Figure 1:**
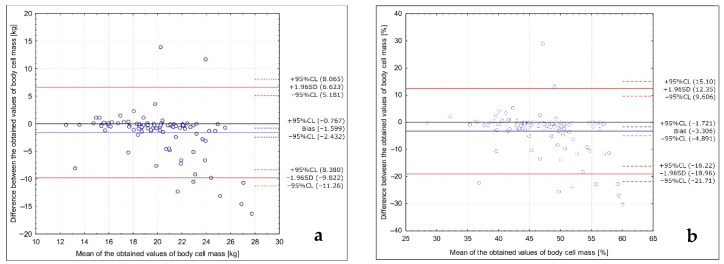
Analysis of the Bland–Altman plots comparing the body cell mass results in the studied group of Caucasian young normal body mass women measured in the follicular phase in various positions (with arms abducted at least 30° and legs abducted at approximately 45°; with arms and legs separated to not touch the body) for the results in kg (**a**) and in the % (**b**).

The analysis of the Bland–Altman plots comparing the body cell mass results in the studied group of Caucasian young normal body mass women measured in the follicular phase in various positions (with arms abducted at least 30° and legs abducted at approximately 45°; with arms and legs separated to not touch the body) is presented in Figure 1. The Bland–Altman indexes of 9% and 9% were observed for the results in kg and in the %, respectively, being interpreted as no agreement between the measurements.

The analysis of the quartile distribution of extracellular water results in the studied group of Caucasian young normal body mass women measured in the follicular phase in various positions (with arms abducted at least 30° and legs abducted at approximately 45°; with arms and legs separated to not touch the body) is presented in Table 4. It was stated that the results for two measurements were compatible (participants classified into the same quartile) for 69% and 53% of the studied group for the results observed in kg and the % of body water, respectively. It corresponded the weighted κ statistics of 0.644 and 0.456, respectively, being interpreted as a substantial and moderate agreement, respectively. It should be indicated that the results not differing (in kg) were associated with a substantial agreement, and those differing (in %) were associated with a moderate agreement. At the same time, while compared with the reference values, 64% of participants were classified into the same category independently from the position (results lower/higher or within the reference values), and while applying a method with arms and legs separated to not touch the body (not recommended position), 31% of participants were underestimated and 5% were overestimated.

**Figure 2:**
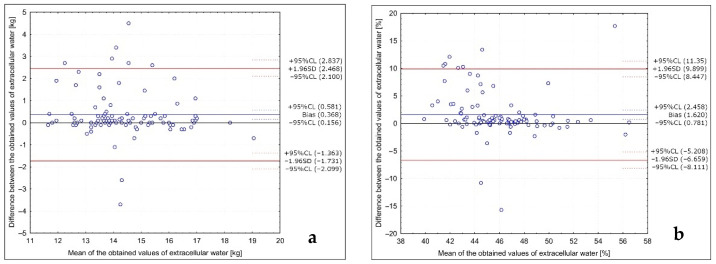
Analysis of the Bland–Altman plots comparing the extracellular water results in the studied group of Caucasian young normal body mass women measured in the follicular phase in various positions (with arms abducted at least 30° and legs abducted at approximately 45°; with arms and legs separated to not touch the body) for the results in kg (**a**) and in the % (**b**).

The analysis of the Bland–Altman plots comparing the extracellular water results in the studied group of Caucasian young normal body mass women measured in the follicular phase in various positions (with arms abducted at least 30° and legs abducted at approximately 45°; with arms and legs separated to not touch the body) is presented in Figure 2. Bland–Altman indexes of 8% and 9% were observed for the results in kg and in the %, respectively, being interpreted as no agreement between the measurements.

The analysis of the quartile distribution of intracellular water results in the studied group of Caucasian young normal body mass women measured in the follicular phase in various positions (with arms abducted at least 30° and legs abducted at approximately 45°; with arms and legs separated to not touch the body) is presented in Table 5. It was stated that the results for two measurements were compatible (participants classified into the same quartile) for 67% and 58% of the studied group for the results observed in kg and the % of body water, respectively. It corresponded the weighted κ statistics of 0.632 and 0.488, respectively, being interpreted as a substantial and moderate agreement, respectively. It should be indicated that the results not differing (in kg) were associated with a substantial agreement, and those differing (in %) were associated with a moderate agreement. At the same time, while compared with the reference values, 66% of the participants were classified into the same category independently from the position (results lower/higher or within the reference values), and while applying a method with arms and legs separated to not touch the body (not recommended position), 6% of participants were underestimated and 28% were overestimated.

**Figure 3:**
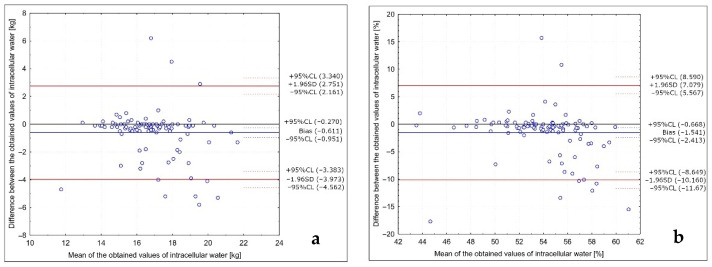
Analysis of the Bland–Altman plots comparing the intracellular water results in the studied group of Caucasian young normal body mass women measured in the follicular phase in various positions (with arms abducted at least 30° and legs abducted at approximately 45°; with arms and legs separated to not touch the body) for the results in kg (**a**) and in the % (**b**).

The analysis of the Bland–Altman plots comparing the intracellular water results in the studied group of Caucasian young normal body mass women measured in the follicular phase in various positions (with arms abducted at least 30° and legs abducted at approximately 45°; with arms and legs separated to not touch the body) is presented in Figure 3. Bland–Altman indexes of 10% and 8% were observed for the results in kg and in the %, respectively, being interpreted as no agreement between the measurements.

The analysis of the quartile distribution of the muscle mass results in the studied group of Caucasian young normal body mass women measured in the follicular phase in various positions (with arms abducted at least 30° and legs abducted at approximately 45°; with arms and legs separated to not touch the body) is presented in Table 6. It was stated that the results for two measurements were compatible (participants classified into the same quartile) for 56% and 51% of the studied group for the results observed in kg and % of the body mass, respectively. It corresponded the weighted κ statistics of 0.488 and 0.472, respectively, being interpreted as a moderate agreement.

**Figure 4:**
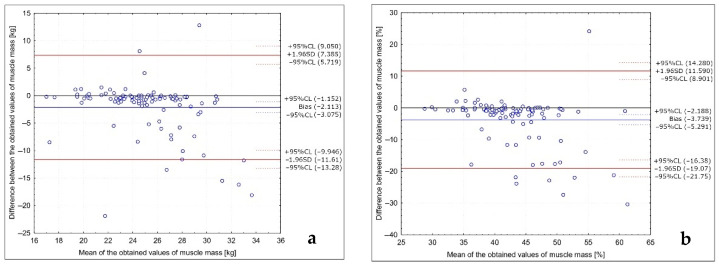
Analysis of the Bland–Altman plots comparing the muscle mass results in the studied group of Caucasian young normal body mass women measured in the follicular phase in various positions (with arms abducted at least 30° and legs abducted at approximately 45°; with arms and legs separated to not touch the body) for the results in kg (**a**) and in the % (**b**).

The analysis of the Bland–Altman plots comparing the muscle mass results in the studied group of Caucasian young normal body mass women measured in the follicular phase in various positions (with arms abducted at least 30° and legs abducted at approximately 45°; with arms and legs separated to not touch the body) is presented in Figure 4. The Bland–Altman indexes of 8% and 8% were observed for the results in kg and in the %, respectively, being interpreted as no agreement between the measurements.

## 4. Discussion

The BIA in healthcare practice may be an effective tool to estimate the body compartments to assess the regular changes in the nutrition status in in-patients and to monitor the nutritional risk in out-patients [35]. However, as indicated based on the conducted study, the applied method of measurement may be crucial, as, without an adequate technique, the bias for the body cell mass, extracellular water, intracellular water, and muscle mass may be significant, so observations of regular changes may be impossible. It results from the fact that, in the presented study, the differences of the measured results dependent on the position were observed for the indicated components of the body composition.

The indicated observations are confirmed by some results by other authors, as Schell & Gross [36] showed a decrease in resistance when the limbs with electrodes at their ends were closer to the body, which influenced the observed changes of the measured results of the body composition. Similar observations were made in studies on the influence of limb length on the obtained impedance values [37], confirming the influence of the applied measurement technique on the obtained results. The above-mentioned studies emphasized that even minor changes associated with limbs (their position or position of electrodes on the limbs) may significantly affect the obtained results of the body composition, which seems to be very important for the methodology of the conducted BIA studies. Moreover, the observations from the presented research indicate a specific influence of the position of the limbs for particular results of the body composition—namely, muscle mass, body cell mass, extracellular water, and intracellular water but not fat mass, fat-free mass, and total body water.

In spite of the fact that a lot of studies use the BIA method to assess only the fat mass, fat-free mass, and total body water, being components not biased significantly, while the position of limbs is changed, the other ones focus exactly on those components in which the conducted study were stated to be biased—namely, the body cell mass, extracellular water, intracellular water, and muscle mass. Those components are important in terms of various groups of individuals, depending on the association with the body conditions. The muscle mass and body cell mass are important for individuals practicing sports [38], as well as malnourished [39], sarcopenia [40], elderly [41], and critically ill patients [42]. At the same time, extracellular water and intracellular water are especially important for individuals experiencing body fluid disturbances, including dehydrated [43], kidney disease [44], and cardiovascular diseases patients [45].

The observation concerning the influence of the position of the limbs on the measured results of the body composition may be explained based on the assumptions defined by Kyle et al. [7], who indicated that the total water content in the body is estimated as the sum of its components from all the body segments, measured in a simplified way as five cylinders—four limbs and the torso. Based on this assumption, it has been observed that the impedance of the arms and legs accounts for 47% and 50% of the total impedance result, respectively, despite the small percentage they represent in the total body weight, and the torso, being about half of the body weight, is responsible for 5–12% of the total body impedance obtained in measurements [46]. It confirms the role of limbs for the measurement and its accuracy, which is of a great importance, as, while the position of the torso is unchangeable, the position of limbs may be changed, and as indicated above, such change may influence the gathered results of the measurements.

In spite of the fact that there are a number of studies carried out while using the recommended method of measurement and controlling carefully the positioning of the limbs [47], it should be noticed that some research studies do not adhere to this recommendation. Some studies present measurements conducted while participants are in a position with arms and legs separated to not touch the body, as is presented in the studies involving patients after bariatric surgery [48], patients during dialysis [49,50], or athletes [51], while the observation of the changes of their body compositions may be crucial for the applied therapy or training, so the inaccurate measurements may be a serious bias. Similarly, such an approach is also chosen in other studies, such as those involving individuals following the Mediterranean diet [52], which also may preclude any reliable conclusion associated with the effect of the applied diet. However, many research studies generally do not describe in detail the applied methodology and regimen of the BIA measurements, which makes it difficult to compare the data between studies and can potentially lead to erroneous measurements [53].

Simplicity and the economic acceptance of the BIA method for body composition estimations have increased the need to unify the protocols and procedures of measurements [48]. If only the recommended body positions of the arms abducted at least 30° and legs abducted at approximately 45° is possible, it should be chosen to guarantee the reliability of the BIA measurements, as no other measurement procedure and no equations developed for other populations are valid and reliable. Therefore, if the research is not using the BIA guidelines for body composition measurements, as described by Kyle et al. [7], it should be justified by specific research goals or by conducted studies indicating that the modified element of the methodology does not affect the obtained measurement results.

In spite of the fact that the conducted study provided novel observations and reported the influence of body positions on the results of the body composition results, some limitations of the study and directions for further research should be listed. First of all, the study was conducted only in a population of young women, so the results should not be extrapolated for the other population groups. At the same time, the measurements were conducted in a specific phase of the menstrual cycle (follicular proliferative phase, chosen as that which is associated with no fluid changes and relatively stable body water content), so the results in the other phases of the menstrual cycle may be also different. Taking this into account, further research conducted in different populations and in the case of women also in different phases of their menstrual cycles are necessary. Moreover, the detailed analysis of the raw results obtained in larger population groups may allow to observe mechanisms of the noted differences, as well as a comparison of the results with the results of the body composition obtained while using the other methods, such as computer tomography, magnetic resonance imaging, or dual-energy X-ray absorptiometry and they would allow deeper conclusions.

## 5. Conclusions

The statistically significant differences dependent on the position were observed for the body cell mass results and muscle mass results, as well as the extracellular and intracellular water results, which were caused by the different results of impedance. While compared with the reference values, the major differences were observed for the extracellular/intracellular water contents, as, while applying a nonrecommended position, the extracellular water content was underestimated and the intracellular water content was overestimated. It may be concluded that the recommended body position of arms abducted at least 30° and legs abducted at approximately 45° should be chosen to ensure the reliability of the BIA measurements, as, while the recommendations of the body position are not followed, the results obtained may be misleading and may not reflect the actual body composition.

## Figures and Tables

**Table 1:** Comparison of the body composition results in the studied group of Caucasian young normal body mass women measured in the follicular phase in various positions (with arms abducted at least 30° and legs abducted at approximately 45°; with arms and legs separated to not touch the body).

Body Composition	Position with Arms Abducted at Least 30° and Legs Abducted at Approximately 45°; *n* = 100				Position with Arms and Legs Separated to Not Touch the Body; *n* = 100				*p* **
	Mean ± SD	Median	Minimum	Maximum	Mean ± SD	Median	Minimum	Maximum	
Fat mass (kg)	16.4 ± 4.0	16.3	5.5	26.9	16.0 ± 4.2	16.2	5.3	26.9	0.5545
Fat mass (%)	27.5 ± 4.5	27.2	13.1	37.5	26.9 ± 4.8	26.8	11.8	37.5	0.3739
Fat-free mass (kg)	42.6 ± 3.5	42.2	33.6	52.6	42.9 ± 3.5	42.6	33.6	52.9	0.5028
Fat-free mass (%)	72.0 ± 6.5	72.6 *	24.9	86.9	72.6 ± 5.5	73.1 *	51.5	88.2	0.6927
Body cell mass (kg)	19.2 ± 3.0	19.2 *	9.2	29.8	20.8 ± 4.4	20.2 *	12.6	35.9	0.0165
Body cell mass (%)	45.1 ± 6.0	45.0	25.7	61.6	48.4 ± 8.7	47.8 *	28.5	75.3	0.0075
Total body water (kg)	31.1 ± 2.6	30.9	24.6	38.5	31.4 ± 2.6	31.2 *	24.6	38.7	0.4495
Total body water (%)	53.1 ± 3.3	53.1	44.8	63.6	53.5 ± 3.5	53.5	44.7	64.5	0.4475
Extracellular water (kg)	14.6 ± 1.5	14.3	11.6	18.7	14.2 ± 1.6	14.0	10.9	19.4	0.0982
Extracellular water (%)	46.8 ± 3.6	46.6 *	38.3	64.2	45.2 ± 4.2	45.3	35.9	57.2	0.0049
Intracellular water (kg)	16.6 ± 1.8	16.6 *	9.4	21.0	17.2 ± 2.1	16.9 *	12.9	23.2	0.0759
Intracellular water (%)	53.2 ± 3.6	53.4 *	35.8	61.7	54.7 ± 4.3	54.6	42.8	68.8	0.0115
Muscle mass (kg)	24.0 ± 3.5	23.9 *	10.8	35.8	26.1 ± 4.8	25.4 *	17.1	42.7	0.0025
Muscle mass (%)	41.0 ± 6.1	40.8 *	27.3	67.2	44.7 ± 8.6	43.8 *	28.9	76.5	0.0011

* Nonparametric distribution (verified while using the Shapiro–Wilk test; *p* ≤ 0.05). ** Data compared while using the Student’s *t*-test (for parametric distributions) and Mann–Whitney *U* test (for nonparametric distributions).

**Table 2:** Analysis of the correlations of the body composition results in the studied group of Caucasian young normal body mass women measured in the follicular phase in various positions (with arms abducted at least 30° and legs abducted at approximately 45°; with arms and legs separated to not touch the body).

Body Composition	*p*	R
Fat mass (kg)	<0.0001	0.9335 *
Fat mass (%)	<0.0001	0.9122 *
Fat-free mass (kg)	<0.0001	0.9059 *
Fat-free mass (%)	<0.0001	0.8613 **
Body cell mass (kg)	<0.0001	0.6103 **
Body cell mass (%)	<0.0001	0.6974 **
Total body water (kg)	<0.0001	0.9052 **
Total body water (%)	<0.0001	0.9119 *
Extracellular water (kg)	<0.0001	0.8645 *
Extracellular water (%)	<0.0001	0.5050 **
Intracellular water (kg)	<0.0001	0.6677 **
Intracellular water (%)	<0.0001	0.5620 **
Muscle mass (kg)	<0.0001	0.6374 **
Muscle mass (%)	<0.0001	0.7152 **

* For analysis of the correlation—Pearson’s correlation coefficient (parametric distribution). ** For analysis of the correlation—Spearman’s rank correlation coefficient (nonparametric distribution).

**Table 3:** Analysis of the quartile distribution of the body cell mass results in the studied group of Caucasian young normal body mass women measured in the follicular phase in various positions (with arms abducted at least 30° and legs abducted at approximately 45°; with arms and legs separated to not touch the body).

		Position with Arms Abducted at Least 30° and Legs Abducted at Approximately 45°				
		Quartile	1st Quartile	2nd Quartile	3rd Quartile	4th Quartile
Position with arms and legs separated to not touch the body	Body cell mass (kg)	1st quartile	20%	1%	1%	3%
		2nd quartile	2%	16%	7%	0%
		3rd quartile	1%	1%	12%	11%
		4th quartile	2%	7%	5%	11%
	Body cell mass (%)	1st quartile	17%	6%	0%	2%
		2nd quartile	3%	15%	7%	0%
		3rd quartile	2%	0%	13%	10%
		4th quartile	3%	4%	5%	13%

**Table 4:** Analysis of the quartile distribution of the extracellular water results in the studied group of Caucasian young normal body mass women measured in the follicular phase in various positions (with arms abducted at least 30° and legs abducted at approximately 45°; with arms and legs separated to not touch the body).

		Position with Arms Abducted at Least 30° and Legs Abducted at Approximately 45°				
		Quartile	1st Quartile	2nd Quartile	3rd Quartile	4th Quartile
Position with arms and legs separated to not touch the body	Extracellular water (kg)	1st quartile	16%	5%	3%	1%
		2nd quartile	6%	17%	1%	1%
		3rd quartile	1%	3%	17%	4%
		4th quartile	2%	0%	4%	19%
	Extracellular water (%)	1st quartile	11%	3%	8%	3%
		2nd quartile	11%	13%	0%	1%
		3rd quartile	1%	8%	12%	4%
		4th quartile	2%	1%	5%	17%

**Table 5:** Analysis of the quartile distribution of intracellular water results in the studied group of Caucasian young normal body mass women measured in the follicular phase in various positions (with arms abducted at least 30° and legs abducted at approximately 45°; with arms and legs separated to not touch the body).

		Position with Arms Abducted at Least 30° and Legs Abducted at Approximately 45°				
		Quartile	1st Quartile	2nd Quartile	3rd Quartile	4th Quartile
Position with arms and legs separated to not touch the body	Intracellular water (kg)	1st quartile	20%	3%	0%	2%
		2nd quartile	0%	19%	5%	1%
		3rd quartile	3%	2%	13%	7%
		4th quartile	2%	1%	7%	15%
	Intracellular water (%)	1st quartile	18%	3%	1%	3%
		2nd quartile	4%	14%	6%	1%
		3rd quartile	0%	0%	15%	10%
		4th quartile	3%	8%	3%	11%

**Table 6:** Analysis of the quartile distribution of the muscle mass results in the studied group of Caucasian young normal body mass women measured in the follicular phase in various positions (with arms abducted at least 30° and legs abducted at approximately 45°; with arms and legs separated to not touch the body).

		Position with Arms Abducted at Least 30° and Legs Abducted at Approximately 45°				
		Quartile	1st Quartile	2nd Quartile	3rd Quartile	4th Quartile
Position with arms and legs separated to not touch the body	Muscle mass (kg)	1st quartile	19%	2%	1%	3%
		2nd quartile	2%	15%	8%	0%
		3rd quartile	0%	3%	11%	11%
		4th quartile	4%	5%	5%	11%
	Muscle mass (%)	1st quartile	15%	10%	0%	0%
		2nd quartile	1%	13%	10%	1%
		3rd quartile	3%	1%	10%	11%
		4th quartile	6%	1%	5%	13%

## Data Availability

Not applicable.

## References

[1] Lemos T., Gallagher D. (2017). Current body composition measurement techniques. Curr. Opin. Endocrinol. Diabetes Obes..

[2] Duren D.L., Sherwood R.J., Czerwinski S.A., Lee M., Choh A.C., Siervogel R.M., Chumlea W.C. (2008). Body composition methods: Comparisons and interpretation. J. Diabetes Sci. Technol..

[3] Jeon K.C., Kim S.-Y., Jiang F.L., Chung S., Ambegaonkar J.P., Park J.-H., Kim Y.-J., Kim C.-H. (2020). Prediction equations of the multifrequency standing and supine bioimpedance for appendicular skeletal muscle mass in Korean older people. Int. J. Environ. Res. Public Health.

[4] Ræder H., Kværner A.S., Henriksen C., Florholmen G., Henriksen H.B., Bøhn S.K., Paur I., Smeland S., Blomhoff R. (2018). Validity of bioelectrical impedance analysis in estimation of fat-free mass in colorectal cancer patients. Clin. Nutr..

[5] Siddiqui N.I., Alam Khan S., Shoeb M., Bose S. (2016). Anthropometric predictors of bio-impedance analysis (BIA) phase angle in healthy adults. J. Clin. Diagn. Res..

[6] Wells J.C.K., Fewtrell M.S. (2006). Measuring body composition. Arch. Dis. Child..

[7] Kyle U.G., Bosaeus I., de Lorenzo A.D., Deurenberg P., Elia M., Gomez J.M., Heitmann B.L., Kent-Smith L., Melchior J.-C., Pirlich M. (2004). Bioelectrical impedance analysis? Part I: Review of principles and methods. Clin. Nutr..

[8] Moissl U.M., Wabel P., Chamney P.W., Bosaeus I., Levin N.W., Bosy-Westphal A., Korth O., Müller M.J., Ellegård L., Malmros V. (2006). Body fluid volume determination via body composition spectroscopy in health and disease. Physiol. Meas..

[9] Lyons-Reid J., Ward L.C., Kenealy T., Cutfield W. (2020). Bioelectrical impedance analysis—An easy tool for quantifying body composition in infancy?. Nutrients.

[10] Walter-Kroker A., Kroker A., Mattiucci-Guehlke M., Glaab T. (2011). A practical guide to bioelectrical impedance analysis using the example of chronic obstructive pulmonary disease. Nutr. J..

[11] Mitra S. (2014). Extracellular hydration, cardiovascular risk, and the interstitium: A three-dimensional view. Kidney Int..

[12] Haverkort E.B., Reijven P.L.M., Binnekade J.M., de van der Schueren M.A.E., Earthman C.P., Gouma D.J., de Haan R.J. (2015). Bioelectrical impedance analysis to estimate body composition in surgical and oncological patients: A systematic review. Eur. J. Clin. Nutr..

[13] Talma H., Chinapaw M.J.M., Bakker B., HiraSing R.A., Terwee C.B., Altenburg T.M. (2013). Bioelectrical impedance analysis to estimate body composition in children and adolescents: A systematic review and evidence appraisal of validity, responsiveness, reliability and measurement error. Obes. Rev..

[14] Lyons-Reid J., Derraik J.G.B., Ward L.C., Tint M., Kenealy T., Cutfield W.S. (2021). Bioelectrical impedance analysis for assessment of body composition in infants and young children—A systematic literature review. Clin. Obes..

[15] Kyle U.G., Bosaeus I., de Lorenzo A.D., Deurenberg P., Elia M., Gómez J.M., Heitmann B.L., Kent-Smith L., Melchior J.-C., Pirlich M. (2004). Bioelectrical impedance analysis—Part II: Utilization in clinical practice. Clin. Nutr..

[16] Reiss J., Iglseder B., Kreutzer M., Weilbuchner I., Treschnitzer W., Kässmann H., Pirich C., Reiter R. (2016). Case finding for sarcopenia in geriatric inpatients: Performance of bioimpedance analysis in comparison to dual X-ray absorptiometry. BMC Geriatr..

[17] Bourdon C., Bartels R.H., Chimwezi E., Kool J., Chidzalo K., Perot L., Brals D., Bandsma R.H., van Hensbroek M.B., Voskuijl W.P. (2021). The clinical use of longitudinal bio-electrical impedance vector analysis in assessing stabilization of children with severe acute malnutrition. Clin. Nutr..

[18] Stapel S.N., Looijaard W.G.P.M., Dekker I.M., Girbes A.R.J., Weijs P.J.M., Oudemans-van Straaten H.M. (2018). Bioelectrical impedance analysis-derived phase angle at admission as a predictor of 90-day mortality in intensive care patients. Eur. J. Clin. Nutr..

[19] Kahraman A., Hilsenbeck J., Nyga M., Ertle J., Wree A., Plauth M., Gerken G., Canbay A.E. (2010). Bioelectrical impedance analysis in clinical practice: Implications for hepatitis C therapy BIA and hepatitis C. Virol. J..

[20] World Health Organization (WHO) Body Mass Index—BMI. https://www.euro.who.int/en/health-topics/disease-prevention/nutrition/a-healthy-lifestyle/body-mass-index-bmi.

[21] Scalfi L., Bedogni G., Marra M., di Biase G., Caldara A., Severi S., Contaldo F., Battistini N. (1997). The prediction of total body water from bioelectrical impedance in patients with anorexia nervosa. Br. J. Nutr..

[22] Salinari S., Bertuzzi A., Mingrone G., Capristo E., Scarfone A., Greco A.V., Heymsfield S.B. (2003). Bioimpedance analysis: A useful technique for assessing appendicular lean soft tissue mass and distribution. J. Appl. Physiol..

[23] Campa F., Levi Micheli M., Pompignoli M., Cannataro R., Gulisano M., Toselli S., Greco G., Coratella G. (2021). The Influence of Menstrual Cycle on Bioimpedance Vector Patterns, Performance, and Flexibility in Elite Soccer Players. Int. J. Sports Physiol. Perform..

[24] Cáceres D.I., Sartor-Messagi M., Rodríguez D.A., Escalada F., Gea J., Orozco-Levi M., Marco E. (2014). Variability in bioelectrical impedance assessment of body composition depending on measurement conditions: Influence of fast and rest. Nutr. Hosp..

[25] Głąbska D., Cackowska K., Guzek D. (2018). Comparison of the body composition of Caucasian young normal body mass women, measured in the follicular phase, depending on the carbohydrate diet level. Medicina.

[26] Hussain R., Levin N., Zhu F., Kappel F., Kotanko P. (2012). Body composition and solute kinetics in hemodialysis patients: A mathematical model. IET Commun..

[27] Jensen B., Braun W., Both M., Gallagher D., Clark P., González D.L., Klückmann K., Bosy-Westphal A. (2020). Configuration of bioelectrical impedance measurements affects results for phase angle. Med. Eng. Phys..

[28] Aandstad A., Holtberget K., Hageberg R., Holme I., Anderssen S.A. (2014). Validity and reliability of bioelectrical impedance analysis and skinfold thickness in predicting body fat in military personnel. Mil. Med..

[29] Langer R.D., Borges J.H., Pascoa M.A., Cirolini V.X., Guerra-Junior G., Gonçalves E.M. (2016). Validity of bioelectrical impedance analysis to estimation fat-free mass in the army cadets. Nutrients.

[30] Dittmar M., Reber H. (2001). New equations for estimating body cell mass from bioimpedance parallel models in healthy older Germans. Am. J. Physiol. Metab..

[31] Fujii K., Ishizaki A., Ogawa A., Asami T., Kwon H., Tanaka A., Sekiya N., Hironaka S. (2017). Validity of using multi-frequency bioelectrical impedance analysis to measure skeletal muscle mass in preschool children. J. Phys. Ther. Sci..

[32] Gonzalez C., Evans J., Smye S., Holland P. (2002). Total body water measurement using bioelectrical impedance analysis, isotope dilution and total body potassium: A scoring system to facilitate intercomparison. Eur. J. Clin. Nutr..

[33] Landis J.R., Koch G.G. (1977). The Measurement of Observer Agreement for Categorical Data. Biometrics.

[34] Myles P., Cui J.I. (2007). Using the Bland–Altman method to measure agreement with repeated measures. Br. J. Anaesth..

[35] Kondrup J., Allison S.P., Elia M., Vellas B., Plauth M. (2003). Educational and clinical practice committee, European Society of Parenteral and Enteral Nutrition (ESPEN). ESPEN guidelines for nutrition screening 2002. Clin. Nutr..

[36] Schell B., Gross R. (2000). The reliability of bioelectrical impedance measurements in the assessment of body composition in healthy adults. Nutr. Rep. Int..

[37] Deurenberg P., Deurenberg-Yap M. (2002). Validation of skinfold thickness and hand-held impedance measurements for estimation of body fat percentage among Singaporean Chinese, Malay and Indian subjects. Asia Pac. J. Clin. Nutr..

[38] Pigłowska M., Kostka T., Drygas W., Jegier A., Leszczyńska J., Bill-Bielecka M., Kwaśniewska M. (2016). Body composition, nutritional status, and endothelial function in physically active men without metabolic syndrome—A 25 year cohort study. Lipids Health Dis..

[39] Mareschal J., Achamrah N., Norman K., Genton L. (2019). Clinical value of muscle mass assessment in clinical conditions associated with malnutrition. J. Clin. Med..

[40] Ribeiro S., Kehayias J.J. (2014). Sarcopenia and the analysis of body composition. Adv. Nutr..

[41] De-Mateo-Silleras B., Camina-Martín M.A., de-Frutos-Allas J.M., de-la-Cruz-Marcos S., Carreño-Enciso L., Redondo-del-Río M.P. (2018). Bioimpedance analysis as an indicator of muscle mass and strength in a group of elderly subjects. Exp. Gerontol..

[42] Fiaccadori E., Morabito S., Cabassi A., Regolisti G. (2014). Body cell mass evaluation in critically ill patients: Killing two birds with one stone. Crit. Care.

[43] Ugras S. (2020). Evaluating of altered hydration status on effectiveness of body composition analysis using bioelectric impedance analysis. Libyan J. Med..

[44] Tai R., Ohashi Y., Mizuiri S., Aikawa A., Sakai K. (2014). Association between ratio of measured extracellular volume to expected body fluid volume and renal outcomes in patients with chronic kidney disease: A retrospective single-center cohort study. BMC Nephrol..

[45] Knudsen N.N., Kjærulff T.M., Ward L.C., Sæbye D., Holst C., Heitmann B.L. (2014). Body water distribution and risk of cardiovascular morbidity and mortality in a healthy population: A prospective cohort study. PLoS ONE.

[46] Dehghan M., Merchant A.T. (2008). Is bioelectrical impedance accurate for use in large epidemiological studies?. Nutr. J..

[47] Bongiovanni T., Mascherini G., Genovesi F., Pasta G., Iaia F.M., Trecroci A., Ventimiglia M., Alberti G., Campa F. (2020). Bioimpedance vector references need to be period-specific for assessing body composition and cellular health in elite soccer players: A brief report. J. Funct. Morphol. Kinesiol..

[48] Khalil S.F., Mohktar M.S., Ibrahim F. (2014). The theory and fundamentals of bioimpedance analysis in clinical status monitoring and diagnosis of diseases. Sensors.

[49] Ismail A.H., Schlieper G., Walter M., Floege J., Leonhardt S. (2019). Knee-to-knee bioimpedance measurements to monitor changes in extracellular fluid in haemodynamic-unstable patients during dialysis. J. Electr. Bioimpedance.

[50] Ismail A.H., Gross T., Schlieper G., Walter M., Eitner F., Floege J., Leonhardt S. (2021). Monitoring transcellular fluid shifts during episodes of intradialytic hypotension using bioimpedance spectroscopy. Clin. Kidney J..

[51] Campa F., Toselli S., Mazzilli M., Gobbo L.A., Coratella G. (2021). Assessment of body composition in athletes: A narrative review of available methods with special reference to quantitative and qualitative bioimpedance analysis. Nutrients.

[52] Barrea L., Muscogiuri G., Macchia P.E., di Somma C., Falco A., Savanelli M.C., Colao A., Savastano S. (2017). Mediterranean diet and phase angle in a sample of adult population: Results of a pilot study. Nutrients.

[53] Brantlov S., Jødal L., Lange A., Rittig S., Ward L.C. (2017). Standardisation of bioelectrical impedance analysis for the estimation of body composition in healthy paediatric populations: A systematic review. J. Med. Eng. Technol..

